# GLUT1 and cerebral glucose hypometabolism in human focal cortical dysplasia is associated with hypermethylation of key glucose regulatory genes

**DOI:** 10.21203/rs.3.rs-4946501/v1

**Published:** 2024-10-17

**Authors:** Chaitali Ghosh, Rosemary Westcott, David Skvasik, Ishant Khurana, Jean Khoury, Ingmar Blumcke, Assam El-Osta, Imad M. Najm

**Affiliations:** Cleveland Clinic; Cleveland Clinic; Cleveland Clinic; Baker IDI Heart and Diabetes Institute; Cleveland Clinic; University Hospitals Erlangen; Baker IDI Heart and Diabetes Institute; Cleveland Clinic

**Keywords:** focal cortical dysplasia, epilepsy, biomarker, glucose transporter-1, DNA methylation

## Abstract

Focal cortical dysplasia (FCD) is recognized as a significant etiological factor in pharmacoresistant intractable epilepsy, linked with disturbances in neurovascular metabolism. Our study investigated regulation of glucose-transporter1 (GLUT1) and cerebral hypometabolism within FCD subtypes. Surgically excised human brain specimens underwent histopathological categorization. A subset of samples (paired with matching blood) was assessed for DNA methylation changes of glucose metabolism-related genes. We evaluated GLUT1, VEGFα, MCT2, and mTOR expression by western blot analysis, measured glucose-lactate concentrations, and established correlations with patients’ demographic and clinical profiles. Furthermore, we investigated the impact of DNA methylation inhibitor decitabine and hypometabolic condition on the uptake of [^3^H]-2-deoxyglucose and ATPase in epileptic brain endothelial cells (EPI-EC). We observed hypermethylation of GLUT1 and glucose metabolic genes in FCD brain/blood samples and could distinguish FCDIIa/b from mMCD, MOGHE and non-lesional types in brain. Low GLUT1 and glucose-lactate ratios corresponded to elevated VEGFα and MCT2 in FCDIIa/b vs non-lesional tissues, independent of age, gender, seizure-onset, or duration of epilepsy. Increased mTOR signaling in FCDIIa/b tissues was evident. Decitabine stimulation increased GLUT1, decreased VEGFα expression, restored glucose uptake and ATPase activity in EPI-ECs and reduced mTOR and MCT2 levels in HEK cells. We demonstrated: 1) hypermethylation of glucose regulatory genes distinguish FCDIIa/b from mMCD, MOGHE and non-lesional types, 2) glucose uptake reduction is due to GLUT1 suppression mediated possibly by a GLUT1-mTOR mechanism; and 3) DNA methylation regulates cellular glucose update and metabolism. Together, these studies may lead to GLUT1-mediated biomarkers, glucose metabolism and identify early intervention strategies in FCD.

## Introduction

Epilepsy is a complex neurological disease, and focal cortical dysplasia (FCD) is a common cause of pharmacoresistant epilepsy [1–4]. Fluoro-deoxy-D-glucose PET radiotracer is routinely used for imaging epileptic foci by localizing areas of focal cerebral hypometabolism [5]. Glucose normally enters the brain by facilitated transport via a glucose transporter (GLUT1/ *SLC2A1*) located on the luminal and abluminal surfaces of the blood-brain barrier (BBB) [6,7]. GLUT1 is responsible for mediating more than 90% of glucose transport across the brain. Notably, its presence is often markedly reduced or absent in FCD regions [8–10,5].

In cortical dysplasia (CD) rodent model brains, we found decreased GLUT1, elevated mammalian target of rapamycin (mTOR) and monocarboxylate transporter 2 (MCT2) expression, and persistent imbalance in glucose-lactate levels upon seizure induction [9]. We further identified increased vessel density which could be due to angiogenesis and BBB dysfunction with decreased cortical tight junction proteins, sustained longer in CD rats post seizure induction. However, the epigenetic regulation by DNA methylation and characterization of GLUT1 pattern in FCD subtypes and the key regulatory proteins responsible for glucose metabolism is presently understudied and unknown.

Decitabine (5-Aza-2′-deoxycytidine, 5Aza) is a nucleoside analogue that incorporates into replicating DNA in place of cytosine, traps and promotes the degradation of DNA methyltransferases, therefore acting as a DNA methylation inhibitor [11,12]. 5Aza, has proven beneficial for cancer therapy as an FDA-approved drug [13–15]. Hence, by assessing 5Aza as a *proof-of-concept*, the DNA demethylation approach may elucidate the functional impact of methylation on the functionality of GLUT1 and the broader glucose metabolic pathway.

This study investigates the regulatory dynamics of GLUT1 and cerebral hypometabolism across various FCD subtypes (such as FCDIIa, FCDIIb, mMCD, and MOGHE) and non-lesional resected tissue, which may pave the way for early diagnostic and therapeutic strategies. We show surgically excised brain tissue (matched with blood samples) from patients with pharmacoresistant epilepsy to enhance the identification of DNA methylation biomarkers for glucose regulation. Additionally, the study assessed the principal glucose regulatory proteins and the glucose/lactate ratios in FCDIIa/b versus non-lesional brain tissues. Correlations were drawn between the metabolic levels of glucose in the brain, protein expressions, and the demographic/clinical attributes of the participants, including age, gender, age at seizure onset, and epilepsy duration. The influence of DNA hypermethylation on these factors is further substantiated through the application of the DNA methylation inhibitor decitabine in EPI-ECs and HEK cells, evaluating GLUT1, VEGFα levels, the uptake pattern of [^3^H]-2-deoxyglucose, ATPase activity, and mTOR signaling pathways.

## Materials and Methods

### Human Subjects

Resected human brain tissues from a de-identified homogeneous population of male and female patients with medically refractory epilepsy (*n* = 57) were histopathologically well-characterized as follows: 1) using the ILAE classification [16] into FCD subtypes: FCDIIa, FCDIIb, mMCD, MOGHE, and non-lesional (Non-FCD on histopathology) neocortical areas, *n* = 54 (Table S1). We utilized a subset of matched brain and blood samples (*n* = 15) under IRB 12–1000. 2) a cohort of paired epileptic and non-epileptic tissues from same brain (*n* = 3, Table S1) pre-characterized through non-invasive evaluation (scalp-video-EEG monitoring, magnetic resonance imaging, and positron emission tomography), invasive recordings (stereo-electroencephalography) and post-resective histopathological examination under IRB 07–322. We obtained written informed consent from each patient under Cleveland Clinic Institutional Review Board-approved protocols (IRB 07–322 and IRB 12–1000).

### DNA methylation

#### Methylation sequencing (Methyl-seq)-

DNA extractions were obtained from a subset of matched brain tissues and blood samples of FCD subtypes (*n* = 15) and compared using methyl-capture [17,18]. Genomic DNA was fragmented by sonication, and 500 ng of genomic DNA was used for methyl-CpG enrichment using MethylMiner (Life Technologies), with a positive control methylated DNA spike-in option. Eluted DNA was then quantified, and 10 ng of this methylated DNA was used to generate Illumina sequencing libraries via DNA Library Preparation Kit [17,18]. Regions of DNA methylation changes was annotated on the UCSC genome browser and expressed as observed differences in DNA methylation for each group. After data quality control, sequenced reads were aligned to the human reference genome and then counted. Profiles of DNA methylation were compared between all pairwise combinations of samples using the MACS peak-calling software. The number of reads aligning to each region (peak) was counted and annotated producing a count matrix (reads/region/sample). Differentially methylated regions of GLUT1/*SLC2A1*, MCT2/*SLC16A7*, *VEGFα*, *MTOR* genes were identified by using edgeR with a generalized linear model.

### Tissue Lysate Preparation and Western Blot

Approximately 50 mg of fresh-frozen human brain cortical tissue (*n* = 49) was lysed with radioimmunoprecipitation assay buffer (Sigma-Aldrich, #R0278) [19,20]. Protein concentration was estimated by the Bradford method. Tissue lysates were separated by 8% or 12% sodium dodecyl sulfate polyacrylamide gel electrophoresis (SDS-PAGE) and transferred to polyvinylidene fluoride membranes by semi-dry transfer, then blocked and probed with the primary antibody followed by the appropriate secondary antibody as previously described [9,20]. Protein expression was normalized by β-actin as loading controls, and the densitometry quantification of the images was performed using ImageJ software. See Methods S1 for details.

### Glucose-Lactate Measurement

Glucose and lactate were measured in human brain tissue samples via a dual-channel immobilized oxidase enzyme analyzer as previously described [9]. The sample supernatants were analyzed for glucose and lactate levels measured in mmol/liter, and the data plotted as glucose/lactate ratio. Details in Methods S1.

### Primary Brain Endothelial Cell and Human Embryonic Kidney Cell Culture

We used primary microvascular endothelial cells (EPI-EC, *n* = 3) derived from brain specimens resected from patients with pharmacoresistant epilepsy as described earlier [19] and Methods S1. Primary control Human Brain Microvascular Endothelial cells (HBMECs) were purchased from Cell Systems (#ACBRI 376). HBMECs were dissociated from normal human brain cortical tissue obtained from healthy donors and characterized by von-Willebrand factor staining. Human Embryonic Kidney (HEK)293 cells were obtained from ATCC (#CRL-1573) [9].

### Cell Treatment with low glucose-high lactate and DNA methylation inhibitor, decitabine/5-Aza-2′-deoxycytidine (5Aza) and Western Blot

Both cell types (EPI-EC and HEK) in the treatment group were cultured separately in 100 mm petri-dishes in complete medium containing 1 mM glucose with 20 mM lactate (Sigma-Aldrich, USA, #71718) for 24 h to mimic the low glucose/high lactate condition observed in the FCD brain. Cells grown in normal DMEM (0 mM lactate) media were used as controls. The DNA methylation inhibitor, 5-Aza-2′-deoxycytidine (5Aza, decitabine) (Sigma Aldrich, USA, #A3656) was evaluated at concentrations of 0, 5, 10, 20 μM for cytotoxicity evaluation [21]. See Methods S1. After 24 h, cells were lysed using RIPA lysis buffer with 1% protease inhibitors for western blot analysis of the target proteins [9]. The antibodies are listed in Table S2.

### Glucose Uptake Assay of [^3^H]-2-deoxyglucose

EPI-EC and HBMEC were treated for 24 h with either 1 mM low glucose and 20 mM lactate or 5 mM normal glucose and 0 mM lactate, with and without 5Aza (10 μM) at 37°C. Then cells were washed three-times with glucose-free media and a final concentration of 1 μCi/ml [^3^H]-2-deoxyglucose and incubated for 15 min. Cells were then washed twice with ice-cold PBS and solubilized in 1% SDS (modified method [22]) and the radioactivity of the sample with scintillation fluid was determined using a Beckmann Coulter counter. Non-specific uptake was measured in the presence of 20 μmol/L cytochalasin B and subtracted from each determination to obtain specific uptake. The protein concentration was determined using the Bradford method. Results were expressed as ^3^[H]-2-deoxyglucose uptake (cpm per μg protein).

### ATPase activity assay

The ATPase activity was measured by detecting the free inorganic phosphate (Pi) using a Pi Per Phosphate Assay kit (Molecular Probes #P22061) using HBMEC, EPI-ECs or and HEK cell lysates [23]. The standards and samples reacted with Amplex Red reagent for 60 min at 37°C using a fluorescence microplate reader, at excitation/530–560 nm and emission/ 590 nm. The levels are represented as μmol of Pi per μg of protein in each specimen. Details in Methods S1.

### Adenylate Kinase (AK)- cytotoxicity assay

Cytotoxicity was assessed in primary ECs (HBMEC and EPI-EC) and HEK by measuring the levels of AK in the supernatant; details in Methods S1.

### Statistical Analysis

Origin Pro 2022 Software was used for data analysis and statistical data interpretation. Student’s t-test was used to compare the DNA methylation in FCD-subtypes and matched blood and brain samples. Either one-way or two-way analysis of variance (ANOVA) was utilized to compare multiple groups, with a Tukey post hoc or Bonferroni test for western blot quantification and the glucose-lactate levels. One-way ANOVA was used for ATPase assay values and glucose uptake. All data are shown as mean ± standard error of the mean (SEM). *P*-value of < 0.05 was considered statistically significant.

## Results

### Hypermethylation of GLUT1 and other key genes responsible for glucose metabolism distinguishes FCDIIa/b from other FCD subtypes in brain

**Figure 1:**
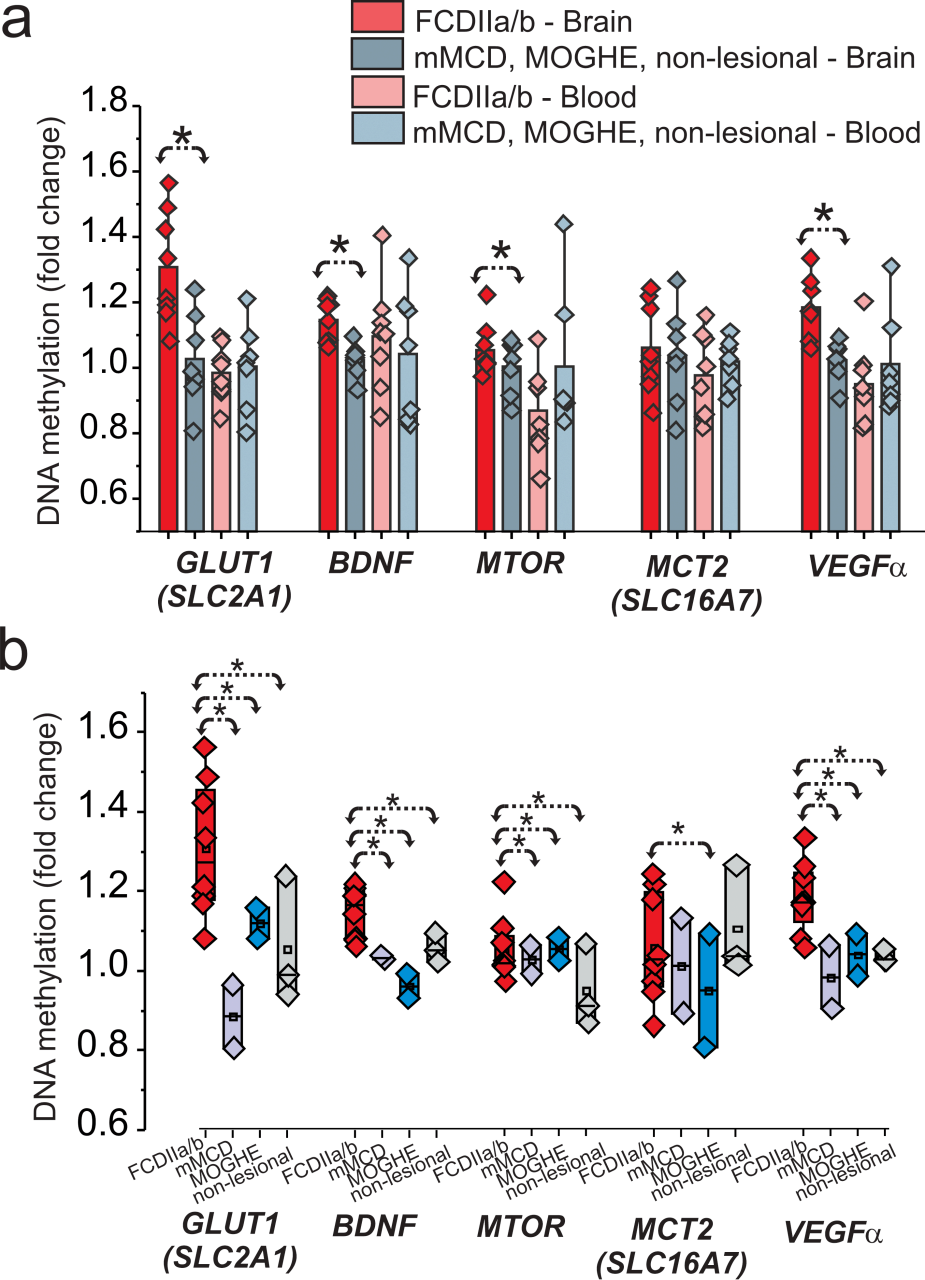
Hypermethylation of GLUT1 and glucose regulatory genes in FCDIIa/b vs other-FCD subtypes in brain. GLUT1 (*SLC2A1*), brain-derived neurotrophic factor (*BDNF*), *MTOR*and MCT2 (*SLC16A7*) brain and matched blood levels (*n*=15) were compared between FCDIIa/b vs other-FCD subtypes, i.e., FCDIIa/b vs mMCD, MOGHE and non-lesional within the patient cohort (**a**) and within FCD subtypes (**b**) in brain tissues. Fold-change using nominal *p*-value from generalized linear model shows gain of methylation in individuals with FCD subtypes for *SLC2A1, BDNF*, and *MTOR* using t-test (*p < 0.05) for comparison

Because recent studies have shown the expression of glucose metabolism pathways are regulated by DNA methylation [18], we assessed its role in brain tissues and matching blood samples obtained from FCD subtypes (FCDIIa/b, mMCD, MOGHE) and non-lesional cases. DNA methylation changes were observed in the brain separate FCDIIa/b from other FCD subtypes, mMCD, MOGHE as well as non-lesional samples (Fig. 1a). A significant (*p < 0.05) fold-change was found for specific targets in brains related to glucose metabolism, *SLC2A1, BDNF, MTOR* and *VEGFα* within FCDIIa/b vs other FCD subtypes (Fig. 1a). No significant difference was seen in the blood (*n* = 15). We found that DNA methylation of GLUT1 (*SLC2A1*) and other key genes (*BDNF, MTOR and VEGFα*) are significantly increased in FCDIIa/b brains (Fig. 1b). These target genes showed a marked difference when compared to mMCD, MOGHE and non-lesional samples (Fig. 1b), thereby categorizing FCDIIa/b from other subtypes using gene methylation as a biomarker.

### Suppressed GLUT1 and low glucose-lactate ratios correspond to elevated VEGFα and MCT2 in FCDIIa and FCDIIb brain tissues, independent of age and gender and not correlated to age of seizure-onset or duration of epilepsy

**Figure 2:**
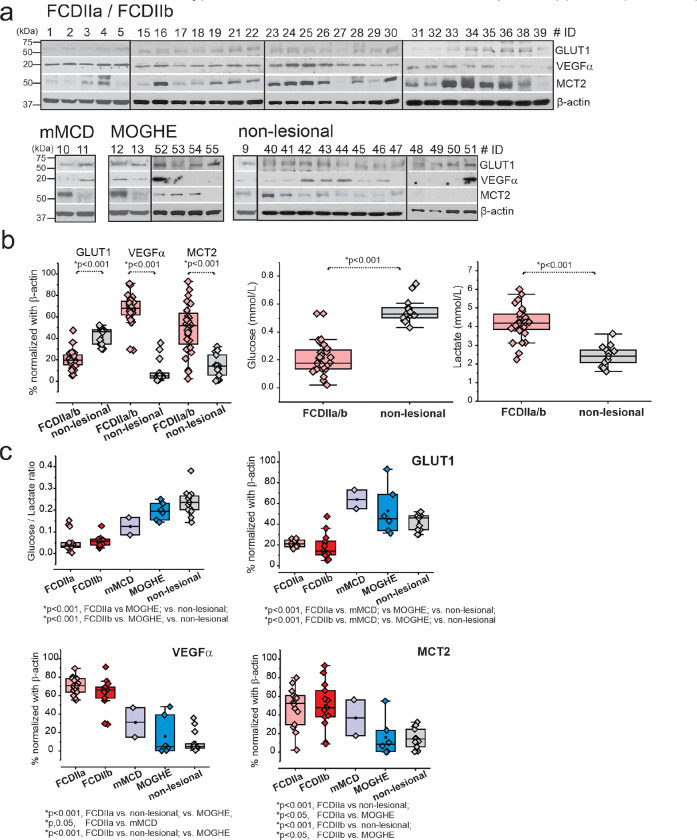
Decreased GLUT1levels, upregulated VEGFα and MCT2 and low glucose and high lactate levels in FCDIIa/b brain tissues. (**a**) Western blot shows decreased GLUT1 levels (55 kDa), and upregulated VEGFα (20 kDa) and MCT2 (53 kDa) in FCDIIa/b, mMCD, MOGHE and non-lesional brain tissues (*n*=49). β-actin (43 kDa) is used as loading control. (**b**) The quantified data of western blot is normalized with loading control, β-actin and plotted to compare FCDIIa/b vs non-lesional. In addition, the same samples analyzed by biochemical assay showed lower glucose and elevated lactate (in mmol/L). Values are mean ± SEM, ***p<0.001, one-way ANOVA. (**c**) Among FCD subtypes a decreased glucose/lactate ratio and GLUT1 protein levels was significant in FCDIIa and FCDIIb compared to other FCD subtypes, mMCD, MOGHE and non-lesional. In contrary, increased levels of VEGFα and MCT2 was noted in the respective FCDIIa/b vs mMCD, MOGHE and non-lesional. The statistical comparison was done by one-way ANOVA with interaction to compare different FCD and non-FCD subtypes, using a Tukey post hoc test. All data are presented as mean ± SEM, and *p<0.05 was statistically significant

Western blot analysis performed on 49 samples indicates a marked decrease in GLUT1 expression, coupled with an increase in VEGFα and MCT2 proteins in FCDIIa/b tissues when compared to mMCD, MOGHE, and non-lesional tissue variants, as depicted in Fig. 2 and Fig S7. Notably, glucose concentrations were significantly reduced (*p < 0.001) in FCDIIa/b, whereas lactate levels were found to be higher relative to non-lesional brain tissue. Furthermore, the observed suppression of GLUT1 and the corresponding low glucose-to-lactate ratios were associated with increased VEGFα levels in FCDIIa/b, as shown in Fig. 2b–c.

An analysis to assess the potential correlation between age or gender and the protein expression in FCDIIa/b versus non-lesional samples was performed. This comparison, illustrated in Fig. S1-S2, spanned various age brackets (0–20; 21–45; >45 years). The results demonstrated no significant differences across age or gender subgroups, with the reduction in GLUT1 and the rise in VEGFα and MCT2 levels in FCDIIa/b remaining consistent across all categories. Similarly, the glucose-to-lactate ratio was consistently lower in FCDIIa/b brain tissues across these age groups compared to non-lesional tissues, as shown in Fig. S1.

Further examination revealed that the alterations in glucose-to-lactate ratios and the levels of GLUT1, VEGFα, and MCT2 proteins in FCDIIa/b versus non-lesional tissues were not influenced by gender (Fig. S2), age of seizure onset (Fig. S3), or the duration of epilepsy (Fig. S4) in either FCDIIa/b or non-lesional subjects. Collectively, these findings underscore a distinct glucose metabolic profile associated with disease pathology and FCD subtype.

### Increased mTOR signaling in FCDIIa/b brains

**Figure 3:**
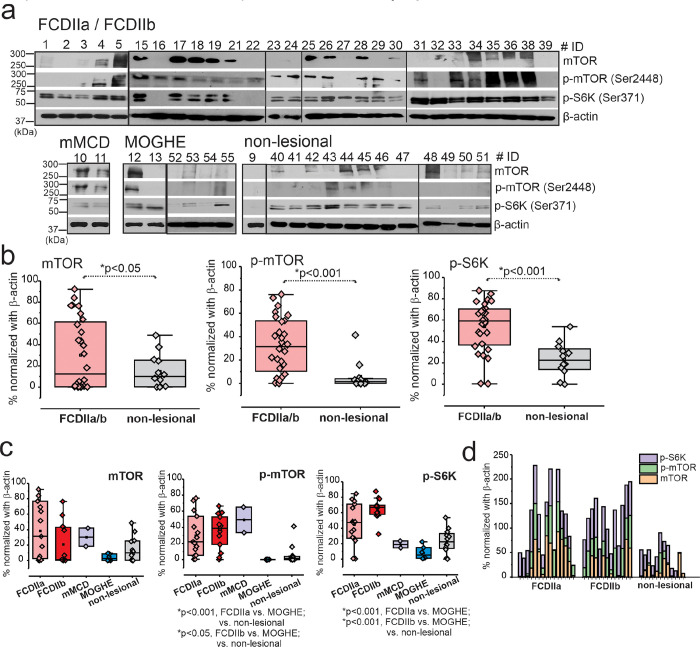
Overall increased mTOR signaling pathway is predominant in dysplastic brains. (**a**) In the FCDIIa/b brain tissues elevated levels of mTOR (289 kDa), p-mTOR (Ser2448) (289 kDa) and p-S6K (Ser371) (70 kDa) by western blot analysis is evident compared to non-lesional, shown by the representative blot (*n*=49). (**b**) The values of these targets analyzed by western blot were normalized with β-actin (43 kDa) and plotted that shows significant increase in mTOR (*p<0.001), p-mTOR (*p<0.001) and p-S6K (*p<0.001). Values are mean ± SEM, ***p<0.001, two-sample t-test for comparison between FCD vs non-lesional brain tissues. Among FCD subtypes, FCDIIa and FCDIIb showed relatively increased mTOR activation compared to other FCD subtypes, mMCD, MOGHE and non-lesional where elevation in mTOR activated, p-mTOR (*p<0.001, FCDIIa vs non-lesional; *p<0.05, FCDIIa vs. MOGHE; *p<0.05, FCDIIb vs. MOGHE/ vs. non-lesional) and p-S6K (*p<0.001, FCDIIa vs MOGHE; *p<0.001, FCDIIb vs vs. MOGHE/ non-lesional) was observed by western blot analysis normalized with β-actin. Pathology comparison in individual levels shows similar trend of increased elevation of mTOR, p-mTOR and p-S6K in FCDIIa and FCDIIb compared to non-lesional. Values are mean ± SEM, ***p<0.001, one-way ANOVA post hoc Tukey test used for comparison within groups

Cortical brain tissues derived from patients with different FCD subtypes revealed significant (*p < 0.05) mTOR overexpression (Fig. 3b) and activated mTOR pathway in FCDIIa/b vs. MOGHE and non-lesional (Fig. 3c). However, no significant difference was observed between FCDIIa/b (*n* = 28) and mMCD (*n* = 2). We observed significant elevation of p-mTOR (*p < 0.001) and p-S6K (*p < 0.001) in FCDIIa/b vs non-lesional brain tissues (Fig. 3 and Fig. S7) and statistically significant increased levels within groups (Fig. 3a–c). Furthermore, the individual comparison of subjects (Fig. 3c–d) clearly distinguishes the level of mTOR, p-mTOR, p-S6K of FCDIIa (*n* = 15) and FCDIIb (*n* = 13) from MOGHE (*n* = 6) and non-lesional (*n* = 12).

### Pharmacological inhibition of DNA methylation increased GLUT1 levels and decreased VEGFα in EPI-ECs with no cellular stress or cytotoxicity

**Figure 4:**
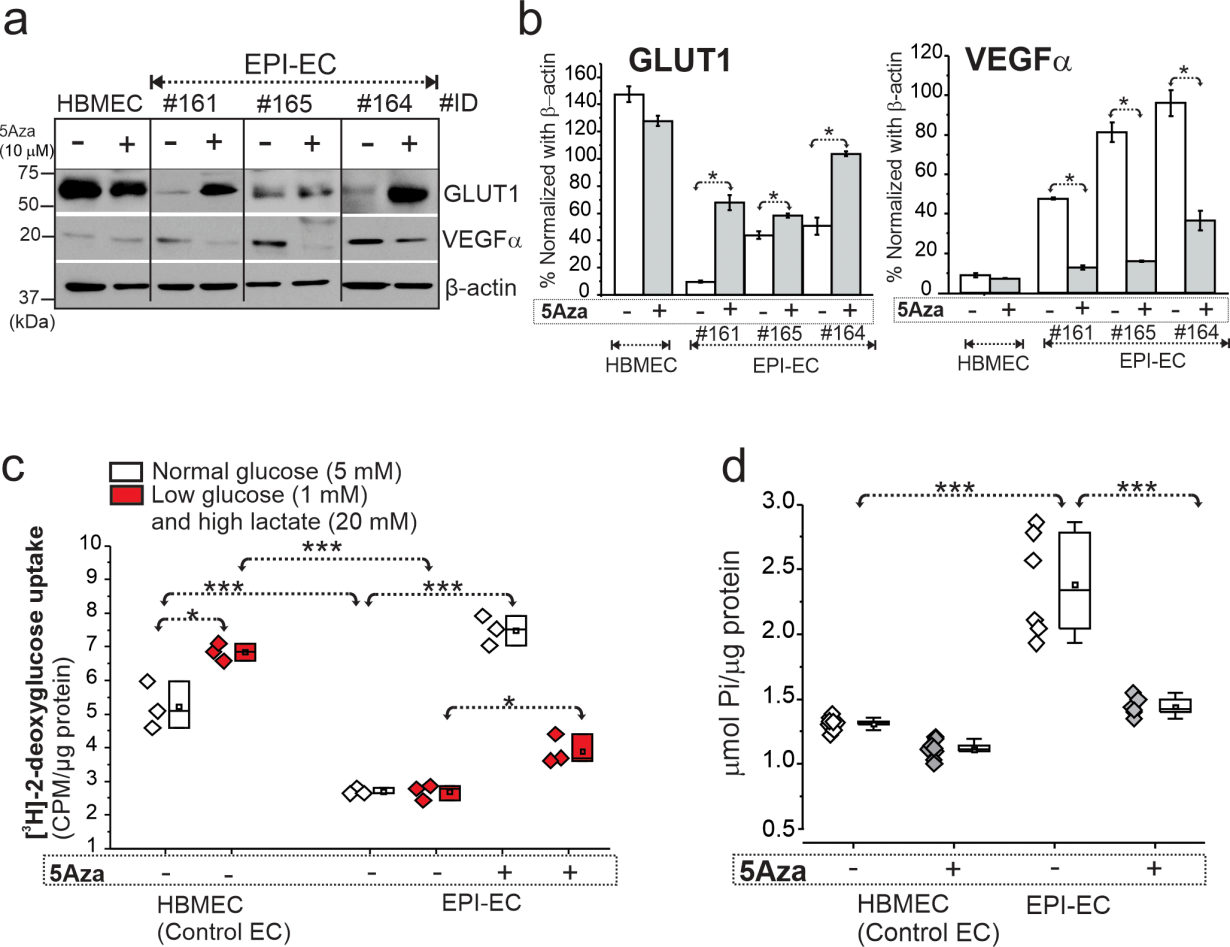
Restoration of GLUT1 and VEGFα levels, glucose uptake function and ATPase activity in FCD EPI-ECs post-DNA methylation inhibition. (**a-b**) DNA methylation inhibitor 5Aza (decitabine) treatment significantly increased GLUT1 levels (*p<0.05) and decreased VEGFα (*p<0.05) in EPI-ECs compared to untreated cells counterpart. Individual EPI-EC from respective patients were denoted by ID #161, #165, #164 and compared with normal EC (HBMEC). Values are mean ± SEM, *p<0.05, two-sample t-test for comparison between conditions, with and without 5Aza treatment. (**c**) Glucose uptake assessed by [^3^H]-2-deoxyglucose radiotracer showed increased uptake in HBMEC under low glucose- high lactate condition compared to normal glucose treatment. However, EPI-EC, under both normal and low glucose-high lactate condition showed non-significant difference. Furthermore, with 5Aza (10 μM) co-treatment in EPI-ECs, the glucose update pattern in cells both under normal (*p<0.001) and low glucose-high lactate (*p<0.05) conditions increased significantly compared to respective untreated 5Aza EPI-EC. Values are mean ± SEM, ***p<0.001, one-way ANOVA with post hoc Tukey test used for group comparisons. (**d**) The increased (*p<0.001) ATPase activity is noted in the EPI-EC compared to HBMEC, which is found to be significantly decreased (*p<0.001) with 5Aza pre-treatment to a level more comparable to control EC. Values are mean ± SEM, ***p<0.001, one-way ANOVA with post hoc Tukey test used for group comparisons

DNA methylation inhibition with decitabine (5Aza) increased GLUT1 levels (*p < 0.05) and decreased VEGFα (*p < 0.05) in FCD EPI-EC compared to control human brain cortical microvascular endothelial cells (HBMEC) which was plotted as percentage normalized with β-actin (Fig. 4a–b). There was no cellular stress or cytotoxicity observed in either primary brain ECs or HEK treated with 5, 10, 20 μM 5Aza (decitabine) when evaluated after 2–24 h (Fig. S5, Fig. S6). See Method section and Table S1 for EPI-EC details.

### Decitabine regulates glucose metabolic functional activity in EPI-EC and HEK

In control HBMEC we observed increased [^3^H]-2-deoxy-glucose uptake under low glucose-high lactate (*p < 0.05) when compared to normoglycemic conditions. In EPI-EC, [^3^H]-2-deoxyglucose uptake was impaired under physiological and low glucose-high lactate condition compared to HBMEC (*p < 0.001). Decitabine restored glucose uptake function of [^3^H]-2-deoxyglucose of EPI-EC under normal glucose (*p < 0.001) and low glucose-high lactate (*p < 0.05) condition compared to untreated EPI-EC, suggesting DNA methylation inhibition regulates the cellular response from dysfunctional glucose uptake observed in EPI-ECs (Fig. 4c). ATPase overactivity in EPI-EC vs HBMEC (*p < 0.001) is also retained back to normal levels (*p < 0.001), post-decitabine stimulation (Fig. 4d). These findings suggest gene methylation regulates glucose-uptake and ATPase activity in EPI-ECs.

**Figure 5:**
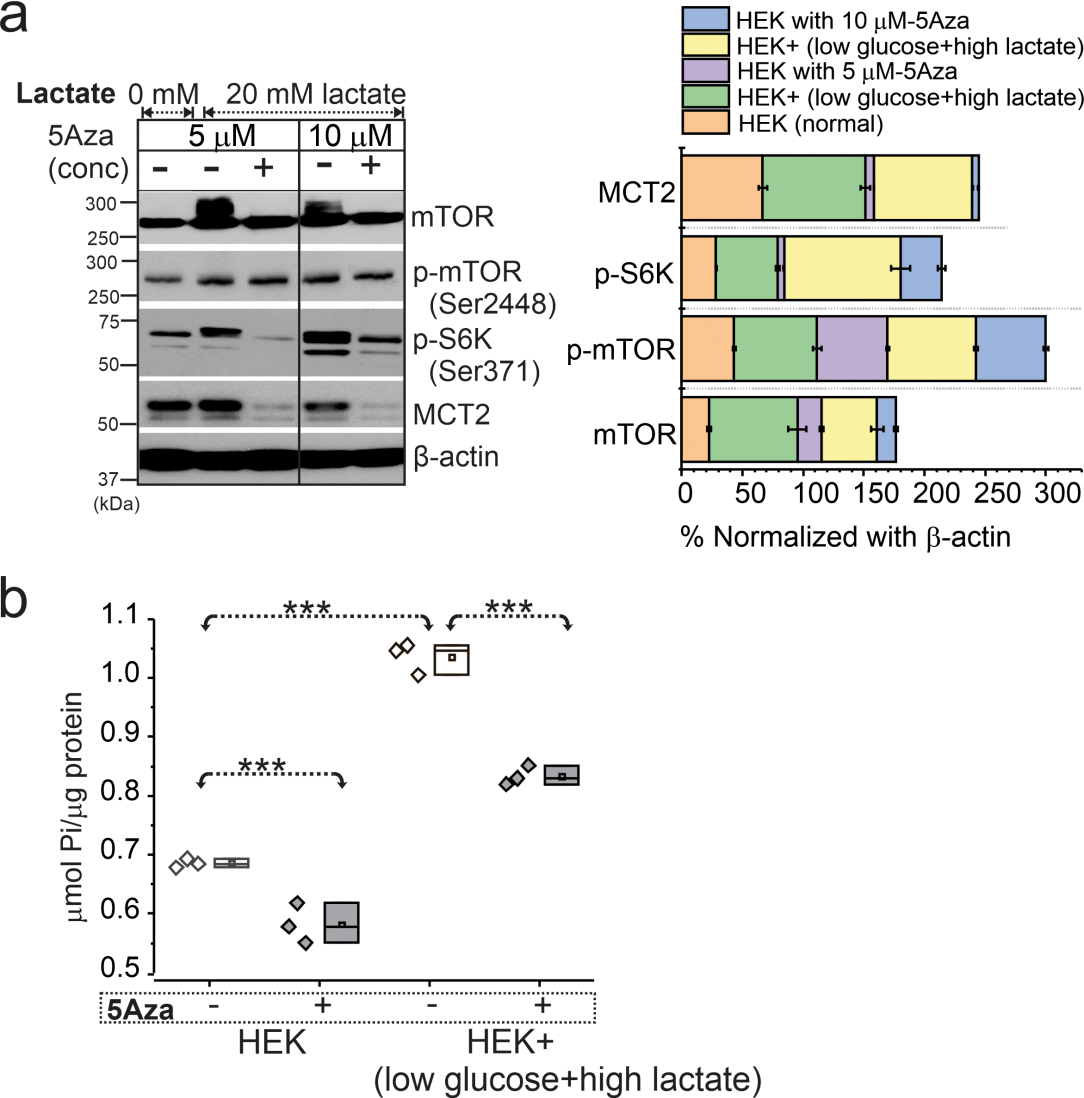
Upregulated mTOR and ATPase activity on HEK cells with low glucose-high lactate state are modulated by DNA methylation. (**a**) HEK cells treated with low glucose-high lactate increased mTOR, p-mTOR, p-S6K and MCT2 levels at 24h. Inhibition of DNA methylation with 5Aza (decitabine) at both 5 and 10 μM reduced mTOR activation and MCT2 levels significantly. The overall trend under different conditions, with and without 5Aza is plotted after normalization with β-actin. (**b**) Upregulated ATPase activity in low glucose-high lactate (*p<0.001) was evident compared to normal (glucose and low lactate state). Further pretreatment of 24 h with 5Aza significantly decreased (*p<0.001) ATPase activity in both these conditions, i.e., low glucose-high lactate and normal glucose treated cells. Values are mean ± SEM, ***p<0.001, one-way ANOVA with post hoc Tukey test used for group comparisons

Similarly, HEK cells known to exhibit several properties of immature neuronal cells when stimulated by hypometabolic condition (Fig. 5a–b) showed upregulated mTOR activity while decitabine significantly reduced mTOR signaling and MCT2 levels. ATPase overactivity in the same HEK cells were evident in low glucose-high lactate condition, compared to normal glucose condition as the hypometabolic-state was maintained. However, such energy demand was restored back to normal ATPase activity (Fig. 5b) in HEK + decitabine group compared to untreated cells, suggesting a role of DNA methylation in regulating mTOR signaling and ATPase in the cells.

## Discussion

The role of DNA methylation on GLUT1 and hypometabolism in focal cortical dysplasia (FCD) remains poorly understood. In this study, we show a differential methylation of key genes involved with glucose uptake and metabolism in epileptic FCDIIa/b. We also compared the influence of methylation on GLUT1 and glucose regulation in FCDIIa/b versus mMCD, MOGHE and non-lesional brain tissues. We further show that pharmacological inhibition of DNA methylation suppresses GLUT1 levels, increases cellular glucose uptake function, ATPase activity and mTOR pathway activation. These results collectively suggest 1) glucose uptake reduction is possibly due to GLUT1 suppression, 2) hypermethylation of *SLC2A1* /GLUT1 and other glucose regulatory genes are specific to FCDIIa/b, and 3) cellular and functional links exist between hypermethylation and GLUT1 downregulation.

Recent studies have shown an integrated pathological and molecular classification of FCD subtypes based on genetic and epigenetic determinants in focal epilepsies [24,25]. In the context of cerebral hypometabolism in this study we have identified that DNA hypermethylation of GLUT1 (*SLC2A1*) and key glucose regulatory genes, including *MTOR, BDNF, VEGFα* and MCT2 (*SLC16A7*) distinguishes FCDIIa/b from other FCD-subtypes (mMCD, MOGHE and non-lesional) in brain samples. The relevance of hypermethylation of GLUT1 and other glucose regulators as potential biomarkers, are indicators of the downstream molecular mechanism unfolding, advancing the clinicopathological understanding in FCD [26,25,16].

The key cell types (e.g., endothelial, astroglial, and neuronal cells), majorly involved in brain parenchymal glucose transport across the blood-brain barrier (BBB), astrocyte-neuronal lactate shuttle and metabolism, contribute to epileptogenesis and seizure propagation [8]. Beside genomic DNA methylation differences between human FCD subtypes, our findings also demonstrated GLUT1 suppression and increased glucose metabolism with low-glucose and high brain lactate levels corresponded to elevated VEGFα in FCDIIa and FCDIIb brain tissues proteins supporting a role of endothelial and astroglial cells in the pathophysiology. Suppression of GLUT1 in cortical dysplasia could contribute to the interictal hypometabolism prevalent in epilepsy [8,10,5]. Such low cerebral glucose levels have also been reported post-severe traumatic brain injury [27,28]. FCDIIa and FCDIIb are known to be the most common malformations of cortical development among children with drug-resistant focal epilepsy [29,16,30]. The findings in this study suggest GLUT1 and glucose metabolism remains significantly altered in FCDIIa and FCDIIb compared to non-lesional tissue samples across various age brackets (0–20; 21–45 and over-45 years old). The suppression of GLUT1 and low glucose-lactate ratio with elevated VEGFα and MCT2 in FCDIIa/b vs non-lesional cases is not age and gender dependent, thereby confirming the metabolic changes observed are specifically due to disease pathology. Despite the trend of GLUT1 suppression and low glucose-lactate ratio and higher VEGFα and MCT2 in FCDIIa/b, we observed no correlation with age of seizure onset or the duration of epilepsy. Such hypometabolic signatures could be valuable for diagnosis regardless of factors such as individual age, gender, age of seizure onset or duration of epilepsy.

Previous reports have shown high-spiking and low-spiking tissues with altered metabolic patterns in human epileptic brain [31]. Low GLUT1 in FCD and glucose deprivation could also predispose neurons to synchronous firing and seizure generation as reported by others [32,10]. Indeed, altered molecular function in human dysplastic versus non-dysplastic brain regions was also recently reported [19]. Protein translation is further enhanced by mTORC1 signaling via phosphorylation of S6 Kinase (p-S6K) to activate the ribosomal protein S6, a component of the 40S ribosomal subunit. Therefore, our observations of mTOR cascade activation in FCD are consistent with previous reports that indicate enhanced mTOR activation in hemimegalencephaly [33,34] and ganglioglioma [35] pathologies and distinguishes tubers from FCD [36,37]. Herein, the p70S6K and p-S6 isoforms in resected FCDIIb specimens, indicating phosphorylated molecules activating downstream targets of the signaling pathways. These reports summarize the molecular events leading to abnormal brain development resulting in mTOR activation evidenced by hyperphosphorylation of mTOR, p-S6K, and S6 proteins. Despite individual variability, we observe significant mTOR activation, particularly the upregulation of p-mTOR and p-S6K in FCDIIa, FCDIIb across subjects compared to non-lesional.

To validate the consequence of these epigenetic changes, the decreased GLUT1 and increased VEGFα levels in FCD EPI-ECs were reversed using the DNA methylation inhibitor, decitabine. Furthermore, glucose uptake dysfunction in EPI-ECs was also rescued within both normal glucose and low-glucose with high-lactate conditions, suggesting a beneficial effect of decitabine on the EPI-ECs, and supporting a role for DNA methylation in regulating glucose trafficking (GLUT1) and angiogenesis (VEGFα) at the BBB. It is also reported that BBB disruption influenced the DNA methylation events via increased expression of noncoding RNA miRNA29b which affects the expression of DNA methyltransferase enzyme (DNMT3b) and matrix metalloproteinases (MMP9). Furthermore, decitabine ameliorates the BBB damage by reducing the expression of miRNA29b [38]. These findings are collectively of great relevance as compromised BBB endothelial function and epilepsy with cortical dysplasia are observed [9,5,39,19]. Future studies could elucidate how BBB leakage induced stress contributes to DNA methylation at the neurovascular unit. These findings corroborate the mechanism of brain metabolic disturbance via DNA methylation which could pave the way towards potential early intervention strategy in FCD.

Signaling of mTOR, p-mTOR (Ser2448) and p-S6K (Ser371) by decitabine was observed in HEK cells under low glucose-high lactate condition. The elevated MCT2 levels decreased following decitabine stimulation suggesting a role for gene hypermethylation in regulating MCT2 levels and mTOR signaling. Lactate is also known to activate the mTORC1 complex in cancer cells [40], and the activation of mTOR signals via HIF1α, initiates VEGF expression in FCDIIb [41]. Higher ATPase activity in cells is similarly downregulated to control levels with decreased phosphate activity following decitabine stimulation. Studies have also reported that lactates operate via negative feedback on neuronal activity by a receptor-mediated mechanism, independent from its intracellular metabolism [42]. Moreover, decitabine was previously shown in fully kindled rat model to increase pentylenetetrazole (a GABA receptor antagonist) induced seizure thresholds to attenuate seizures, and to suppress epileptogenesis [43,44].

## Conclusions

This study highlights a role for DNA hypermethylation on GLUT1 suppression and glucose regulation, which is elevated in human FCDIIa/b subtype as an epigenetic signature related to glucose metabolism. Furthermore, we show DNA methylation activates key glucose regulatory pathway protein targets and cellular function linked to glucose uptake, ATPase activity and thereby improving functional efficacy. Collectively these findings pave a foundation for clinical intervention and diagnostic strategies of biomarker in FCD related to GLUT1 and hypometabolism anomalies.

## Data Availability

The data that support the findings of this study are available from the corresponding author upon reasonable request. Some data may not be made available because of privacy or ethical restrictions.

## References

[1] Gaillard WD, White S, Malow B, Flamini R, Weinstein S, Sato S, Kufta C, Schiff S, Devinsky O, Fazilat S, (1995) FDG-PET in children and adolescents with partial seizures: role in epilepsy surgery evaluation. Epilepsy Res 20 (1):77–84. doi:10.1016/0920-1211(94)00065-5 7713062

[2] Guerrini R, Duchowny M, Jayakar P, Krsek P, Kahane P, Tassi L, Melani F, Polster T, Andre VM, Cepeda C, Krueger DA, Cross JH, Spreafico R, Cosottini M, Gotman J, Chassoux F, Ryvlin P, Bartolomei F, Bernasconi A, Stefan H, Miller I, Devaux B, Najm I, Giordano F, Vonck K, Barba C, Blumcke I (2015) Diagnostic methods and treatment options for focal cortical dysplasia. Epilepsia 56 (11):1669–1686. doi:10.1111/epi.13200 26434565

[3] Halac G, Delil S, Zafer D, Isler C, Uzan M, Comunoglu N, Oz B, Yeni SN, Vatankulu B, Halac M, Ozkara C (2017) Compatibility of MRI and FDG-PET findings with histopathological results in patients with focal cortical dysplasia. Seizure 45:80–86. doi:10.1016/j.seizure.2016.11.024 27960132

[4] WHO (2019) WHO. Epilepsy. Fact Sheet 2019. . WHO. doi:http://www.who.int/mediacentre/factsheets/fs999/en/index.html

[5] Tenney JR, Rozhkov L, Horn P, Miles L, Miles MV (2014) Cerebral glucose hypometabolism is associated with mitochondrial dysfunction in patients with intractable epilepsy and cortical dysplasia. Epilepsia 55 (9):1415–1422. doi:10.1111/epi.12731 25053176

[6] Duelli R, Kuschinsky W (2001) Brain glucose transporters: relationship to local energy demand. News Physiol Sci 16:71–76. doi:10.1152/physiologyonline.2001.16.2.71 11390952

[7] Veys K, Fan Z, Ghobrial M, Bouche A, Garcia-Caballero M, Vriens K, Conchinha NV, Seuwen A, Schlegel F, Gorski T, Crabbe M, Gilardoni P, Ardicoglu R, Schaffenrath J, Casteels C, De Smet G, Smolders I, Van Laere K, Abel ED, Fendt SM, Schroeter A, Kalucka J, Cantelmo AR, Walchli T, Keller A, Carmeliet P, De Bock K (2020) Role of the GLUT1 Glucose Transporter in Postnatal CNS Angiogenesis and Blood-Brain Barrier Integrity. Circ Res 127 (4):466–482. doi:10.1161/CIRCRESAHA.119.316463 32404031 PMC7386868

[8] Fei Y, Shi R, Song Z, Wu J (2020) Metabolic Control of Epilepsy: A Promising Therapeutic Target for Epilepsy. Front Neurol 11:592514. doi:10.3389/fneur.2020.592514 33363507 PMC7753014

[9] Ghosh C, Myers R, O’Connor C, Williams S, Liu X, Hossain M, Nemeth M, Najm IM (2022) Cortical Dysplasia in Rats Provokes Neurovascular Alterations, GLUT1 Dysfunction, and Metabolic Disturbances That Are Sustained Post-Seizure Induction. Mol Neurobiol. doi:10.1007/s12035-021-02624-2 PMC9018620 35084654

[10] Stafstrom CE (2006) Epilepsy: a review of selected clinical syndromes and advances in basic science. J Cereb Blood Flow Metab 26 (8):983–1004. doi:10.1038/sj.jcbfm.9600265 16437061

[11] Palii SS, Van Emburgh BO, Sankpal UT, Brown KD, Robertson KD (2008) DNA methylation inhibitor 5-Aza-2’-deoxycytidine induces reversible genome-wide DNA damage that is distinctly influenced by DNA methyltransferases 1 and 3B. Mol Cell Biol 28 (2):752–771. doi:10.1128/MCB.01799-07 17991895 PMC2223421

[12] Rocha MA, Veronezi GMB, Felisbino MB, Gatti MSV, Tamashiro W, Mello MLS (2019) Sodium valproate and 5-aza-2’-deoxycytidine differentially modulate DNA demethylation in G1 phase-arrested and proliferative HeLa cells. Sci Rep 9 (1):18236. doi:10.1038/s41598-019-54848-x 31796828 PMC6890691

[13] Issa JP (2005) Optimizing therapy with methylation inhibitors in myelodysplastic syndromes: dose, duration, and patient selection. Nat Clin Pract Oncol 2 Suppl 1:S24–29. doi:10.1038/ncponc0355 16341237

[14] Karahoca M, Momparler RL (2013) Pharmacokinetic and pharmacodynamic analysis of 5-aza-2’-deoxycytidine (decitabine) in the design of its dose-schedule for cancer therapy. Clin Epigenetics 5 (1):3. doi:10.1186/1868-7083-5-3 23369223 PMC3570332

[15] Silverman LR, Demakos EP, Peterson BL, Kornblith AB, Holland JC, Odchimar-Reissig R, Stone RM, Nelson D, Powell BL, DeCastro CM, Ellerton J, Larson RA, Schiffer CA, Holland JF (2002) Randomized controlled trial of azacitidine in patients with the myelodysplastic syndrome: a study of the cancer and leukemia group B. J Clin Oncol 20 (10):2429–2440. doi:10.1200/JCO.2002.04.117 12011120

[16] Najm I, Lal D, Alonso Vanegas M, Cendes F, Lopes-Cendes I, Palmini A, Paglioli E, Sarnat HB, Walsh CA, Wiebe S, Aronica E, Baulac S, Coras R, Kobow K, Cross JH, Garbelli R, Holthausen H, Rossler K, Thom M, El-Osta A, Lee JH, Miyata H, Guerrini R, Piao YS, Zhou D, Blumcke I (2022) The ILAE consensus classification of focal cortical dysplasia: An update proposed by an ad hoc task force of the ILAE diagnostic methods commission. Epilepsia 63 (8):1899–1919. doi:10.1111/epi.17301 35706131 PMC9545778

[17] Harikrishnan KN, Chow MZ, Baker EK, Pal S, Bassal S, Brasacchio D, Wang L, Craig JM, Jones PL, Sif S, El-Osta A (2005) Brahma links the SWI/SNF chromatin-remodeling complex with MeCP2-dependent transcriptional silencing. Nat Genet 37 (3):254–264. doi:10.1038/ng1516 15696166

[18] Khurana I, Kaipananickal H, Maxwell S, Birkelund S, Syreeni A, Forsblom C, Okabe J, Ziemann M, Kaspi A, Rafehi H, Jorgensen A, Al-Hasani K, Thomas MC, Jiang G, Luk AO, Lee HM, Huang Y, Thewjitcharoen Y, Nakasatien S, Himathongkam T, Fogarty C, Njeim R, Eid A, Hansen TW, Tofte N, Ottesen EC, Ma RC, Chan JC, Cooper ME, Rossing P, Groop PH, El-Osta A (2023) Reduced methylation correlates with diabetic nephropathy risk in type 1 diabetes. J Clin Invest 133 (4). doi:10.1172/JCI160959 PMC9927943 36633903

[19] Westcott R, Chung N, Ghosh A, Ferguson L, Bingaman W, Najm IM, Ghosh C (2022) Glucocorticoid Receptor beta Isoform Predominates in the Human Dysplastic Brain Region and Is Modulated by Age, Sex, and Antiseizure Medication. Int J Mol Sci 23 (9). doi:10.3390/ijms23094940 PMC9099578 35563330

[20] Williams S, Hossain M, Ferguson L, Busch RM, Marchi N, Gonzalez-Martinez J, Perucca E, Najm IM, Ghosh C (2019) Neurovascular Drug Biotransformation Machinery in Focal Human Epilepsies: Brain CYP3A4 Correlates with Seizure Frequency and Antiepileptic Drug Therapy. Mol Neurobiol 56 (12):8392–8407. doi:10.1007/s12035-019-01673-y 31243719 PMC6854685

[21] Gailhouste L, Liew LC, Hatada I, Nakagama H, Ochiya T (2018) Epigenetic reprogramming using 5-azacytidine promotes an anti-cancer response in pancreatic adenocarcinoma cells. Cell Death Dis 9 (5):468. doi:10.1038/s41419-018-0487-z 29700299 PMC5920091

[22] Jiang X, Kenerson H, Aicher L, Miyaoka R, Eary J, Bissler J, Yeung RS (2008) The tuberous sclerosis complex regulates trafficking of glucose transporters and glucose uptake. Am J Pathol 172 (6):1748–1756. doi:10.2353/ajpath.2008.070958 18511518 PMC2408433

[23] Ghosh C, Westcott R, Perucca E, Hossain M, Bingaman W, Najm I (2022) Cytochrome P450-mediated antiseizure medication interactions influence apoptosis, modulate the brain BAX/Bcl-X(L) ratio and aggravate mitochondrial stressors in human pharmacoresistant epilepsy. Front Pharmacol 13:983233. doi:10.3389/fphar.2022.983233 36515436 PMC9441576

[24] Baldassari S, Ribierre T, Marsan E, Adle-Biassette H, Ferrand-Sorbets S, Bulteau C, Dorison N, Fohlen M, Polivka M, Weckhuysen S, Dorfmuller G, Chipaux M, Baulac S (2019) Dissecting the genetic basis of focal cortical dysplasia: a large cohort study. Acta Neuropathol 138 (6):885–900. doi:10.1007/s00401-019-02061-5 31444548 PMC6851393

[25] Kobow K, Ziemann M, Kaipananickal H, Khurana I, Muhlebner A, Feucht M, Hainfellner JA, Czech T, Aronica E, Pieper T, Holthausen H, Kudernatsch M, Hamer H, Kasper BS, Rossler K, Conti V, Guerrini R, Coras R, Blumcke I, El-Osta A, Kaspi A (2019) Genomic DNA methylation distinguishes subtypes of human focal cortical dysplasia. Epilepsia 60 (6):1091–1103. doi:10.1111/epi.14934 31074842 PMC6635741

[26] Kobow K, El-Osta A, Blumcke I (2013) The methylation hypothesis of pharmacoresistance in epilepsy. Epilepsia 54 Suppl 2:41–47. doi:10.1111/epi.12183 23646970

[27] Plummer MP, Notkina N, Timofeev I, Hutchinson PJ, Finnis ME, Gupta AK (2018) Cerebral metabolic effects of strict versus conventional glycaemic targets following severe traumatic brain injury. Crit Care 22 (1):16. doi:10.1186/s13054-017-1933-5 29368635 PMC5784688

[28] Vespa PM, McArthur D, O’Phelan K, Glenn T, Etchepare M, Kelly D, Bergsneider M, Martin NA, Hovda DA (2003) Persistently low extracellular glucose correlates with poor outcome 6 months after human traumatic brain injury despite a lack of increased lactate: a microdialysis study. J Cereb Blood Flow Metab 23 (7):865–877. doi:10.1097/01.WCB.0000076701.45782.EF 12843790

[29] Blumcke I, Spreafico R, Haaker G, Coras R, Kobow K, Bien CG, Pfafflin M, Elger C, Widman G, Schramm J, Becker A, Braun KP, Leijten F, Baayen JC, Aronica E, Chassoux F, Hamer H, Stefan H, Rossler K, Thom M, Walker MC, Sisodiya SM, Duncan JS, McEvoy AW, Pieper T, Holthausen H, Kudernatsch M, Meencke HJ, Kahane P, Schulze-Bonhage A, Zentner J, Heiland DH, Urbach H, Steinhoff BJ, Bast T, Tassi L, Lo Russo G, Ozkara C, Oz B, Krsek P, Vogelgesang S, Runge U, Lerche H, Weber Y, Honavar M, Pimentel J, Arzimanoglou A, Ulate-Campos A, Noachtar S, Hartl E, Schijns O, Guerrini R, Barba C, Jacques TS, Cross JH, Feucht M, Muhlebner A, Grunwald T, Trinka E, Winkler PA, Gil-Nagel A, Toledano Delgado R, Mayer T, Lutz M, Zountsas B, Garganis K, Rosenow F, Hermsen A, von Oertzen TJ, Diepgen TL, Avanzini G, Consortium E (2017) Histopathological Findings in Brain Tissue Obtained during Epilepsy Surgery. N Engl J Med 377 (17):1648–1656. doi:10.1056/NEJMoa1703784 29069555

[30] Najm IM, Tilelli CQ, Oghlakian R (2007) Pathophysiological mechanisms of focal cortical dysplasia: a critical review of human tissue studies and animal models. Epilepsia 48 Suppl 2:21–32. doi:10.1111/j.1528-1167.2007.01064.x 17571350

[31] Wu HC, Dachet F, Ghoddoussi F, Bagla S, Fuerst D, Stanley JA, Galloway MP, Loeb JA (2017) Altered metabolomic-genomic signature: A potential noninvasive biomarker of epilepsy. Epilepsia 58 (9):1626–1636. doi:10.1111/epi.13848 28714074 PMC5910657

[32] Bazzigaluppi P, Ebrahim Amini A, Weisspapir I, Stefanovic B, Carlen PL (2017) Hungry Neurons: Metabolic Insights on Seizure Dynamics. Int J Mol Sci 18 (11). doi:10.3390/ijms18112269 PMC5713239 29143800

[33] Aronica E, Boer K, Baybis M, Yu J, Crino P (2007) Co-expression of cyclin D1 and phosphorylated ribosomal S6 proteins in hemimegalencephaly. Acta Neuropathol 114 (3):287–293. doi:10.1007/s00401-007-0225-6 17483958

[34] Ljungberg MC, Bhattacharjee MB, Lu Y, Armstrong DL, Yoshor D, Swann JW, Sheldon M, D’Arcangelo G (2006) Activation of mammalian target of rapamycin in cytomegalic neurons of human cortical dysplasia. Ann Neurol 60 (4):420–429. doi:10.1002/ana.20949 16912980

[35] Boer K, Troost D, Timmermans W, van Rijen PC, Spliet WG, Aronica E (2010) Pi3K-mTOR signaling and AMOG expression in epilepsy-associated glioneuronal tumors. Brain Pathol 20 (1):234–244. doi:10.1111/j.1750-3639.2009.00268.x 19371356 PMC8094642

[36] Baybis M, Yu J, Lee A, Golden JA, Weiner H, McKhann G 2nd, Aronica E, Crino PB (2004) mTOR cascade activation distinguishes tubers from focal cortical dysplasia. Ann Neurol 56 (4):478–487. doi:10.1002/ana.20211 15455405

[37] Miyata H, Chiang AC, Vinters HV (2004) Insulin signaling pathways in cortical dysplasia and TSC-tubers: tissue microarray analysis. Ann Neurol 56 (4):510–519. doi:10.1002/ana.20234 15455398

[38] Kalani A, Kamat PK, Familtseva A, Chaturvedi P, Muradashvili N, Narayanan N, Tyagi SC, Tyagi N (2014) Role of microRNA29b in blood-brain barrier dysfunction during hyperhomocysteinemia: an epigenetic mechanism. J Cereb Blood Flow Metab 34 (7):1212–1222. doi:10.1038/jcbfm.2014.74 24802332 PMC4083388

[39] Vezzani A (2005) VEGF and seizures: cross-talk between endothelial and neuronal environments. Epilepsy Curr 5 (2):72–74. doi:10.1111/j.1535-7597.2005.05209.x 16059441 PMC1176313

[40] Benjamin D, Hall MN (2019) Lactate jump-starts mTORC1 in cancer cells. EMBO Rep 20 (6). doi:10.15252/embr.201948302 PMC6549024 31133599

[41] Boer K, Troost D, Spliet WG, van Rijen PC, Gorter JA, Aronica E (2008) Cellular distribution of vascular endothelial growth factor A (VEGFA) and B (VEGFB) and VEGF receptors 1 and 2 in focal cortical dysplasia type IIB. Acta Neuropathol 115 (6):683–696. doi:10.1007/s00401-008-0354-6 18317782 PMC2386160

[42] Bozzo L, Puyal J, Chatton JY (2013) Lactate modulates the activity of primary cortical neurons through a receptor-mediated pathway. PLoS One 8 (8):e71721. doi:10.1371/journal.pone.0071721 23951229 PMC3741165

[43] Boison D, Rho JM (2020) Epigenetics and epilepsy prevention: The therapeutic potential of adenosine and metabolic therapies. Neuropharmacology 167:107741. doi:10.1016/j.neuropharm.2019.107741 31419398 PMC7220211

[44] Williams-Karnesky RL, Sandau US, Lusardi TA, Lytle NK, Farrell JM, Pritchard EM, Kaplan DL, Boison D (2013) Epigenetic changes induced by adenosine augmentation therapy prevent epileptogenesis. J Clin Invest 123 (8):3552–3563. doi:10.1172/JCI65636 23863710 PMC3726154

